# The association between skin auto-fluorescence of palmoplantar sites and microvascular complications in Asian patients with type 2 diabetes mellitus

**DOI:** 10.1038/s41598-018-24707-2

**Published:** 2018-04-20

**Authors:** Jong Jin Kim, Bosu Jeong, Yongin Cho, Mi-hyang Kwon, Yong-ho Lee, Uk Kang, Eun Seok Kang

**Affiliations:** 1 Russia Science Seoul Center, Advanced Medical Device Research Division, Korea Electrotechnology Research Institute, Seoul, Republic of Korea; 2 Division of Endocrinology and Metabolism, Department of Internal Medicine, Yonsei University College of Medicine, Seoul, Republic of Korea; 3 Diabetes Center, Severance Hospital, Yonsei University Health System, Seoul, Republic of Korea; 4 Dinstitute of Endocrine Research, Yonsei University College of Medicine, Seoul, Republic of Korea

## Abstract

Skin auto-fluorescence (SAF) has generated broad interest about the prospects for non-invasive advanced glycation end product assessment and its direct interplay with the development of microvascular complications, but clinical application of the existing SAF measuring of non-palmoplantar sites in non-Caucasian subjects with dark skin type is still controversial. Here, we tested the diabetic complication screening performance of a novel SAF measuring system in Asian type 2 diabetes mellitus (T2DM) subjects. A total of 166 Korean patients with T2DM were enrolled in this study and palmoplantar SAF was measured by a newly developed transmission-geometry noninvasive optical system. We found that transmitted SAF values of palmoplantar sites, 1st dorsal interossei muscles of the hand, in a complication group were significantly higher than in a non-complication group while no differences were observed between the two groups in reflected SAF of non-palmoplantar sites. The transmitted SAF values of palmoplantar sites were dramatically increased in subjects with multiple complications and were tightly correlated with the duration of microvascular complications. In conclusion, the SAF measurement in the palmoplantar sites with a non-invasive transmission-geometry optical system provided better microvascular complication screening performance compared to the SAF measurement of non-palmoplantar sites specifically in Asian T2DM subjects.

## Introduction

The blood glucose homeostasis is tightly regulated throughout the body and is critically important to ensure sufficient glucose supply for metabolic demands. In patients with diabetes mellitus, sustained defects in the blood-glucose regulatory system, however, induce long-standing hyperglycemic state, lead to a persistent non-enzymatic reaction between reducing sugar and amino groups of proteins, lipids and nucleic acids and eventually form irreversible advanced glycation end-products (AGEs)^1,2^. The hyperglycemia-induced AGE formation and accumulation in tissues is involved in diverse pathophysiological processes and plays a pivotal role in the progression of different microvascular complications including nephropathy, retinopathy and neuropathy, which are rapidly expanding serious health problems^3–5^.

Nonenzymatic collagen cross-linking occurs throughout the body as a consequence of AGE pathogenesis^6^. Multiple research groups have demonstrated that skin auto-fluorescence (SAF) mainly emanating from cumulative AGE-mediated collagen cross-linking with slow-turnover is correlated to a wide variety of long-term complications especially in patients with DM^7–9^. Unlike invasive AGE quantification in human tissue samples, the SAF has generated broad interest about the prospects for non-invasive AGE assessment and earlier detection of microvascular complications^10^. Despite the general consensus that SAF may be a promising biomarker for the development and progression of AGE-mediated microvascular complications, several confounding factors should be further considered before its clinical application. SAF is conventionally measured on the volar side of the forearms where dark pigmentation density is relatively higher than in palmoplantar sites^11–13^. Among several SAF confounding factors, dark pigmentation in non-palmoplantar areas may affect SAF measurement and interrupt the coupling between measured SAF and past long-term glycemic status^14^. Thus, extra attention even in Caucasian subjects should be necessary to perform SAF measurement on the volar side of the forearms with less dark pigmentation.

In the previous study, we reported that non-invasive SAF measurement of the palmoplantar sites, 1st dorsal interossei muscles of the hand with transmission-geometry provided a superior diagnostic performance of abnormal glucose tolerance (AGT) in Korean subjects even compared to blood sample based AGT diagnosis or to SAF reflected from non-palmoplantar sites^15^. Here, we tested the diabetic complication screening performance of SAF transmitted through the 1st dorsal interossei muscles with a newly developed optical system in Korean patients with type 2 diabetes mellitus (T2DM).

## Results

**Figure 1:**
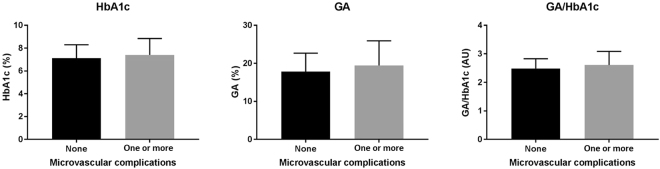
Glycated Serum Protein levels and Microvascular Complications. Statistical comparison of HbA1c, GA and GA/HbA1c ratio between a non-complication group and a complication group. Error bars represent standard deviation.

Of a total of 166 Korean patients with T2DM, the prevalence of diabetic microvascular complications was 66.2% (n = 110); specifically, 36.1% (n = 60), 23.5% (n = 39), and 41.6% (n = 69) of subjects had retinopathy, neuropathy, and nephropathy, respectively. In the physical examination, significant differences between a complication group and a non-complication group were not clearly observed with respect to sex, age, body mass index, and systolic blood pressure and diastolic blood pressure. There were no statistical differences in HbA1c, glycated albumin (GA) and the ratio of GA and HbA1c (GA/HbA1c) between two groups (Fig. 1 and Table 1). On the other hand, complication group had significantly longer T2DM duration and had higher risks of hypertension or hyperlipidemia with no difference in fasting plasma glucose and glycated albumin levels (Table 1). The proportions of angiotensin converting inhibitors (ACEi), angiotensin receptor blocker (ARB) and statin user were higher in the group with complications. Patients’ characteristics are shown in Table 1.

**Table 1:** Baseline characteristics of patients according to the complication status.

	Subjects with DM complication (n = 110)	Subjects without DM complication (n = 56)	P value
Age, years	60.9 ± 11.5	59.3 ± 10.7	0.368
Sex, male	72 (65.5)	39 (69.6)	0.588
Body mass index, kg/m^2^	25.4 ± 4.2	25.5 ± 4.1	0.914
Waist circumference, cm	89.9 ± 9.6	89.1 ± 9.7	0.610
SBP, mmHg	123.7 ± 15.1	121.2 ± 12.3	0.287
DBP, mmHg	71.4 ± 9.3	73.9 ± 10.8	0.121
DM duration, years	12.7 ± 9.1	8.6 ± 6.4	**<0**.**001**
HbA1c, %	7.4 ± 1.4	7.1 ± 1.2	0.218
HbA1c, mmol/mol	57 ± 16	54 ± 13	0.218
Glycated Albumin, %	19.3 ± 5.7	17.7 ± 5.0	0.107
GA/HbA1c (AU)	2.4 ± 0.9	2.3 ± 0.8	0.103
FPG, mg/dL	134.5 ± 44.2	127.2 ± 32.0	0.224
Total cholesterol, mg/dL	158.8 ± 33.9	160.5 ± 34.8	0.768
HDL-cholesterol, mg/dL	43.7 ± 10.7	45.5 ± 10.9	0.144
Triglyceride, mg/dL	148.7 ± 79.3	132.9 ± 57.1	0.304
LDL-cholesterol, mg/dL	85.5 ± 27.4	88.4 ± 29.3	0.517
UACR (mg/g)	477.1 ± 1430.0	10.5 ± 9.9	0.001
eGFR (ml/min/1.73 m^2^)	77.0 ± 30.8	88.1 ± 19.1	0.005
SAF-R (AU)	2.8 ± 1.1	3.0 ± 1.2	**0**.**306**
SAF-T (AU)	6.4 ± 2.5	5.1 ± 1.7	**<0**.**001**
Hypertension	78 (70.9)	26 (46.4)	**0**.**002**
ACEIs or ARBs	71 (64.5)	19 (33.9)	**<0**.**001**
CCBs	39 (35.5)	17 (30.4)	**0**.**316**
Beta-blockers	26 (23.6)	12 (21.4)	**0**.**455**
Diuretics	20 (18.2)	6 (10.7)	**0**.**152**
Hyperlipidemia	86 (78.2)	35 (62.5)	**0**.**032**
Statins	75 (68.2)	29 (51.8)	**0**.**030**
Ezetimibe	15 (13.6)	3 (5.4)	**0**.**083**
Fenofibrate	7 (6.4)	2 (3.6)	**0**.**362**
Retinopathy	60 (36.1)	—	
Neuropathy	39 (23.5)	—	
Nephropathy	69 (41.6)	—	

Data are expressed as the mean ± standard deviation (SD) for continuous variables and percentage (%) for categorical variables. SBP, systolic blood pressure; DBP, diastolic blood pressure; AU, arbitrary unit; FPG, fasting plasma glucose; HDL, high-density lipoprotein; LDL, low-density lipoprotein; UACR, urine albumin-to-creatinine ratio; eGFR, estimated glomerular filtration rate; SAF-R, reflected skin auto-fluorescence on the volar side of the forearms; SAF-T, transmitted skin auto-fluorescence in the 1^st^ interossei muscle of the hand; ACEIs, angiotensin converting enzyme inhibitors; ARBs, angiotensin receptor blockers and CCBs, calcium channel blockers.

**Figure 2:**
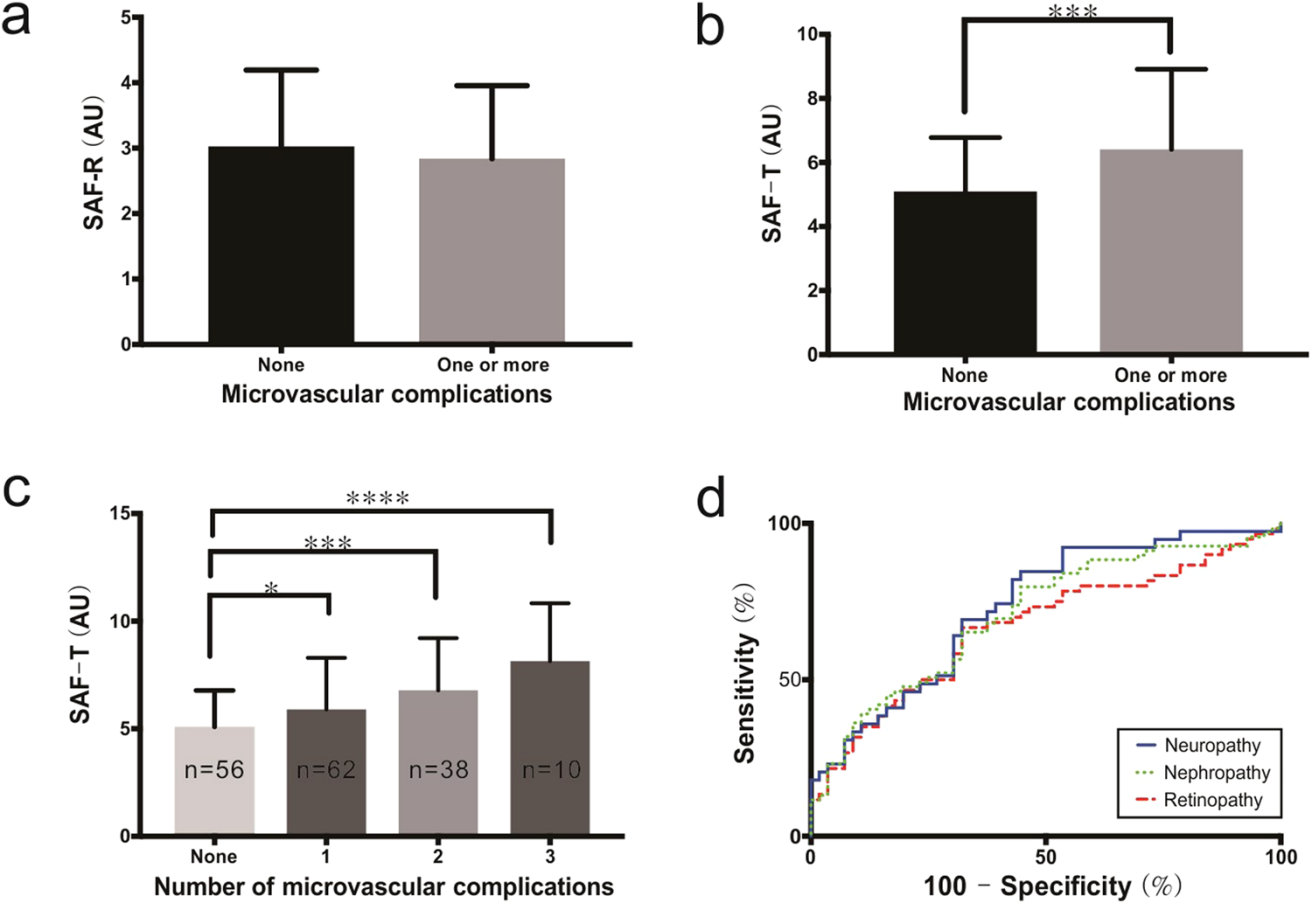
SAF and Microvascular Complications. (**a**) Statistical comparison of reflected SAF values on the volar side of the forearms (SAF-R) between a complication group and a non-complication group. (**b**) Statistical comparison of transmitted SAF values in the 1st dorsal interossei muscles of the hand (SAF-T) between a complication group and a non-complication group. (**c**) SAF-T values with respect to the number of microvascular complications. (**d**) SAF-T utilized receiver operating characteristic curves regarding different microvascular complications. Error bars represent standard deviation. (Asterisks *, **, ***, and **** indicate p < 0.05, p < 0.01, p < 0.001, and p < 0.0001, respectively).

There were no differences between a complication group and a non-complication group with respect to SAF values measured on the volar side of the forearm (SAF-R, Fig. 2a). On the other hand, measured SAF values of the 1^st^ dorsal interossei muscles of the hand (SAF-T) in a complication group were found to be significantly higher than in a non-complication group (6.41 ± 2.50 A.U. vs. 5.10 ± 1.68 A.U., P = 0.0005, Fig. 2b). Intriguingly, the SAF-T was significantly increased in subjects with multiple complications; one complication (n = 62), 5.23 ± 2.40 A.U.; two complications (n = 38), 6.40 ± 2.42 A.U.; three complications (n = 10), 7.78 ± 2.69 A.U., P < 0.000 1 (Fig. 2c).

Pearson’s correlation coefficient revealed that the SAF-T values were positively correlated with the duration of T2DM in each subject (r = 0.2439; P = 0.0015) but independent to the prevalence of hypertension or hyperlipidemia and to all the other physical parameters. The correlation of the duration of T2DM with the number of microvascular complications in each subject was significant (r = 0.2984, P < 0.0001). In univariate logistic regression, microvascular diabetic complications were associated with SAF-T, DM duration, hypertension and hyperlipidemia. Multivariate logistic regression showed that SAF-T (odds ratio (OR), 1.31; 95% confidence interval [CI], 1.08–1.56; P = 0.005) and hypertension (OR, 2.17; 95% CI, 1.04–4.52; P = 0.04) were independent risk factors for complications. When the purpose of a multivariable regression analysis is to explain the individual effects of the predictors on an outcome variable, potential multicollinearity between the variables should be further investigated. Multicollinearity between SAF-T and diabetes duration was assessed using variation influence factor (VIF). VIF was 1.026 which excluded a serious problem with multicollinearity^16^. In addition, the area under curve (AUC) came out to be 0.661 which is statistically significant (P < 0.001) in that SAF-T can predict the diabetic microvascular complication shown by this method (Fig. 2d). An SAF-T cutoff of 4.83 A.U. produced the maximum Youden index, a sum of sensitivity (72.73%) and specificity (55.36%) by Receiver operating characteristic (ROC) analysis. At the SAF-T cutoff of 4.83 A.U., sensitivity for the presence of one or more complications, or diabetic microvascular complication (retinopathy, neuropathy, and nephropathy) was calculated. It predicted one complication at a sensitivity of 61.29% with an AUC of 0.588, two complications at a sensitivity of 86.84% with an AUC of 0.734, and three complications at a sensitivity of 90.00% with an AUC of 0.843. The sensitivity and the AUC significantly increased with an increasing number of complications. Regarding diabetic microvascular complications, patients with retinopathy, neuropathy, and nephropathy were identified with a sensitivity of 71.67%, 84.62%, and 79.71%, respectively. An AUC for determining retinopathy was 0.669, neuropathy was 0.732, and nephropathy was 0.707, respectively. An ROC analysis revealed that the AUC for SAF had a high predictive ability for the presence of neuropathy among diabetic vascular complications.

## Discussions

The glycated serum proteins that are predominant Amadori-type glycation products have long been considered as a determinant marker of the glycemic status over the previous two to four weeks^17,18^. Moreover, several clinical studies have reported that glycated serum protein levels; especially glycated serum albumin levels are tightly associated with the onset of vascular complications by inducing inflammatory mediators in the vascular wall^6,19^. However, it is still controversial whether a single measurement of glycated serum protein level can be an effective predictor of diabetic complication onset in clinical practice. In the present study, we found that there were no significant differences between groups with and without any developing diabetic complications with respect to glycated serum protein levels including HbA1c and GA levels or to HbA1c/GA ratio. Furthermore, we found HbA1c and GA levels were uncoupled to the duration of T2DM that has been well described as a diabetic complication co-factor. Thus, our data confirms that temporal glycated serum protein levels in patients with T2DM do not reflect the long-term glycemic history that correlates with the severity of diabetic complications.

Unlike short-lived glycation serum proteins, AGEs have been implicated as a long-term memory of glycemic status^20,21^. In patients with chronic hyperglycemia, AGE formation and accumulation of proteins or tissues and its direct interplay with multiple pathogenic processes have long been considered as a major threatening factor of diabetic complication onset^3^. Once AGE pathogenesis triggers macro- or micro-angiopathy, impaired cellular AGE defense system as well as enhanced oxidative stress, in turn, promote further AGE accumulation. In clinical settings, the assessment of tissue AGE overload was first conducted with immunolabeling methods in tissue biopsy specimens including retinal blood walls or peripheral nerves^22–24^. Although the direct assessment of AGE quantification from specific target tissues advanced our knowledge on the direct link between AGE accumulation and the severity of diabetic complications, such invasive AGE assessment technique still hampers clinical application because of high surgical difficulties and costs.

In the previous study, the skin biopsies were obtained from the same measurement site in which label-free skin fluorescence measurement was conducted and several AGEs were quantified with multimodal methods^25,26^. The study emphasized that skin fluorescence is closely associated with dermal AGE levels and the development of non-invasive label-free skin fluorescence measuring system enables reliable assessment of the degree of AGE pathogenesis throughout the body. During the last decade, multiple research groups have demonstrated that skin fluorescence on the volar side of the forearms is closely associated with the progression of different microvascular complications in patients with diabetes mellitus up to Fitzpatrick skin phototype class IV^14,27,28^. This tight coupling is owing to temporal relationship between skin fluorescence intensity and past long-term glycemic status. However, elevated dermal chromophores in subjects with dark skin type can result in inaccurate skin fluorescence assessment and eventually lead to unreliable outcomes^29^.

In this study, we found that whereas SAF of the volar side of the forearm with a reflection-geometry optical system remained unassociated with the diabetic complications in Korean patients with T2DM, SAF measured in the 1st dorsal interossei muscle of the hand with a newly developed transmission-geometry optical system was strongly associated with the onset of progressive or sustained diabetic complications in Korean patients with T2DM, in which more than 10% of subjects are generally classified as Fitzpatrick skin phototype class V^30,31^. The mechanistic explanation for these intriguing results needs to be further considered. Since skin fluorophore detection sensitivity highly depends on the efficiency of excitation light delivery, the effective launching of incident light to the desired skin layers is critical to ensure accurate measurement of skin fluorescence.

In a reflection-geometry system, the majority of incident light is directly reflected back by the surface of volar side of the forearm^32^. The reduced delivery of incident light in the desired tissue layers can produce poor detection sensitivity of skin fluorophore density. On the other hand, in the diffused transmission-geometry system, effective delivery of SAF excitation light owing to significant reduction of direct light reflection from skin surface as well as anisotropic diffusion of photons can potentiate a better SAF measuring performance than systems involving reflection-geometry^15^. In addition to better excitation light delivery in transmission-geometry system, unlike the volar side of the forearm on which existing optical systems detect skin fluorescence, the secretion of dickkopf –related protein 1 (DKK1) in palmoplantar human skin suppresses melanocyte function and growth^33^. The melanocyte density in the 1st dorsal interossei is much less than that found on the volar side of the forearm. Thus, skin auto-fluorescence measurement in the 1st dorsal interossei muscle can be less affected by dermal chromophore density. Overall, SAF detection with a transmission-geometry system in the palmoplantar sites potentiates a better SAF measuring performance than systems involving reflection-geometry.

The pathophysiological processes triggered AGE accumulation can affect a wide variety of human tissues and a substantial number of T2DM patients gradually develop multiple complications^3,34^. Thus, various types of medical examination are highly necessary for the complete diagnosis of all of these complications. However, multiple examinations on patients without the corresponding complications are costly. Therefore, if one can diagnose the severity of diabetic complications beforehand, the time and cost of the overall testing can be reduced. Here, we found that skin auto-fluorescence measured in the 1st dorsal interossei muscle dramatically increased in patients with multiple microvascular complications, which suggests that skin auto-fluorescence can be a marker for the severity of diabetic complications.

Furthermore, the duration of abnormal glucose metabolism in patients with T2DM was significant longer in patients with multiple sustained diabetic complications. It is important to note that skin auto-fluorescence measured with a newly developed transmission-geometry optical system reflects the duration of T2DM. Because the duration of T2DM is a critical co-factor of the onset of diabetic complications in patients with T2DM^35^ but unlike type 1 diabetes mellitus accurate assessment of T2DM duration often faces formidable obstacles. Non-invasive and reliable monitoring of T2DM duration holds a great potential for the setting of preemptive and patient-specific strategy against progressive diabetic complications.

In conclusion, palmoplantar SAF levels measured with a transmission-geometry optical system were tightly associated with the duration and the severity of microvascular complications in Korean patients with T2DM and the new modality of SAF detection could be used as regular monitoring methods for microvascular complications even in non-Caucasian subjects with dark skin type.

## Methods

This study enrolled T2DM patients who were aged 20 years or older and did not have any disease or defects of the hand skin. Participants were treated with oral hypoglycemic agent or insulin at the Diabetes Center at Severance Hospital from May to July 2015. Informed consent was obtained from the participants, all of whom agreed to get their SAF measured. T2DM was diagnosed according to the International Classification of Diseases, 10th revision. Among 200 patients, 34 subjects without clinical parameters of HbA1c and glycated albumin within 1 week of SAF measurement were excluded. As a result, 166 patients were finally enrolled in this study. This study was carried out in accordance with the principles of the Declaration of Helsinki as revised in 2000. The study protocol was approved by the Ethics Committee of Yonsei University Health System, Severance Hospital (1-2012-0042 and 1-2015-0014). In all subjects, physical examinations and plasma glucose measurements were performed. The microvascular complications including retinopathy, nephropathy, and neuropathy were diagnosed with conventional screening methods. Briefly, urinary albumin/creatinine ratio (UACR) was calculated using results of urine microanalysis results (Hitachi 7180, Tokyo, Japan). The presence of diabetic nephropathy was defined as UACR of 30 mg/g or more. The presence of diabetic retinopathy was defined as the existence of either proliferative or non-proliferative retinal disease in dilated fundus examination by an ophthalmologist or a previous history of laser photocoagulation therapy. Nerve conducting velocity (NCV) was performed to confirm the presence of neuropathy. Abnormalities in sensory or motor neuronal velocity were defined as neuropathy. In addition, the presence of neuropathy was determined according to the existence of typical symptoms if diabetic neuropathy or history of medication for diabetic neuropathy. A group of subjects who were screened positive to any complication screening in general practice was defined as a complication group; otherwise defined as a non-complication group. The classification of hyperlipidemia and hypertension was made with previous diagnosis.

In each subject, reflected SAF on the volar side of the forearms and transmitted SAF in the 1st dorsal interossei muscle of the hand were measured with a currently developed multi-geometry optical system as described previously^15^. Briefly, in the reflection-geometry system, the volar side of the forearm was sequentially illuminated with dual wavelength beams. The SAF and reference optical signals were collected and focused to a spectrometer (AvaSpec-2048-EDU-VIS Spectrometer; Avantes, Apeldoorn, Netherlands) for spectrum analysis. In the transmission-geometry system, the upper surface of the 1st dorsal interossei muscle of a hand was illuminated by dual wavelength beams. The SAF emitted by the excitation beam and transmitted reference optical signals were collected by a multimode optical fiber located on the other side of muscle and focused to a spectrometer (AvaSpec-2048-EDU-VIS Spectrometer; Avantes) for spectrum analysis. The ratio of average SAF to non-fluorescent reference signals is used for SAF calibration, as previously reported^12^. The system was fully evaluated and calibrated with customized fluorescent test samples (Labsphere, INC., North Sutton, NH) which have the same excitation (center wavelength: 370 nm) and emission (center wavelength: 440 nm) spectra to AGE-related fluorophores.

Among all T2DM subjects, a chi-square test was used for sex difference and a Mann-Whitney test was used for difference in DM duration between positive and negative subjects. All other statistical comparisons were made with an unpaired student’s t-test. One-way analysis of variance test was conducted to compare SAF values varying with the number of associated complications. Pearson’s and Spearman’s correlation coefficient were calculated for assessment of correlation between quantitative and qualitative variables, respectively. Univariate logistic regression analyses were performed in order to determine which variables among age, sex, DM duration, body mass index, waist circumference, systolic blood pressure, diastolic blood pressure, high-density lipoprotein, low-density lipoprotein and triglyceride, SAF-T determined the presence or development of microvascular disease. Factors with a P value less than 0.05 in the univariate model were introduced in to the multivariate logistic regression to estimate the odds ratio and 95% confidence interval. VIF was used to check multicollinearity of variables. ROC curves were plotted to evaluate the diabetic complication screen performance of a newly developed transmission-geometry SAF measuring system. The optimal cutoff values for detecting a diabetic microvascular complication were identified using the maximum of the Youden index.
